# Assessment of the phytochemical constituents and antioxidant activity of a bloom forming microalgae *Euglena tuba*

**DOI:** 10.1186/0717-6287-47-24

**Published:** 2014-06-04

**Authors:** Dipankar Chaudhuri, Nikhil Baban Ghate, Shampa Deb, Sourav Panja, Rhitajit Sarkar, Jayashree Rout, Nripendranath Mandal

**Affiliations:** Division of Molecular Medicine, Bose Institute, P-1/12 CIT Scheme VIIM, Kolkata, 700054 India; Department of Ecology and Environmental Science, Assam University, Silchar, 788011 Assam India

**Keywords:** Euglenoid, Phytochemicals, Free radical scavenging, Antioxidant, Reducing power

## Abstract

**Background:**

Unstable generation of free radicals in the body are responsible for many degenerative diseases. A bloom forming algae *Euglena tuba* growing abundantly in the aquatic habitats of Cachar district in the state of Assam in North-East India was analysed for its phytochemical contents, antioxidant activity as well as free radical scavenging potentials.

**Results:**

Based on the ability of the extract in ABTS^•+^ radical cation inhibition and Fe^3+^ reducing power, the obtained results revealed the prominent antioxidant activity of the algae, with high correlation coefficient of its TEAC values to the respective phenolic and flavonoid contents. The extract had shown its scavenging activity for different free radicals and 41.89 ± 0.41 μg/ml, 5.83 ± 0.07 μg/ml, 278.46 ± 15.02 μg/ml and 223.25 ± 4.19 μg/ml were determined as the IC_50_ values for hydroxyl, superoxide, nitric oxide and hypochlorous acid respectively, which are lower than that of the corresponding reference standards. The phytochemical analysis also revealed that the phenolics, flavonoids, alkaloids, tannins and carbohydrates are present in adequate amount in the extract which was confirmed by HPLC analysis.

**Conclusions:**

The results showed that 70% methanol extract of the algae possesses excellent antioxidant and free radical scavenging properties.

## Background

*Euglena tuba* (Carter) (Family – *Euglenaceae*) is an unicellular euglenozoa distributed in most aquatic bodies all over India throughout the year, with the prominent seasonal algal bloom of *Euglena* occurring in the winter season. In general, algal bloom is an incidental event which tends to occur when a period of calm weather coincides with nutrient enrichment and excess buoyancy of the population of algal cells. Early reports of red coloured euglenozoa blooms from India were recognised as *Euglena tuba*, *E. orientalis* and *E. haematodes* [1]. Recent researches have demonstrated that a number of bloom forming algae like *Dunaliella*, *Chlorella*, *Chlamydomonas*, *Ochromonas*, *Spirulina* and *Euglena* have attracted immense attention for their significant antioxidant properties [2–5]. Different species of *Euglena* has been screened for their simultaneous production of more than a single antioxidant compound like β-carotene, vitamin C and vitamin E, rendering it a promising dietary supplement [6]. Since *Euglena* being non-toxic and does not pose any threat to the ecosystem, it presents an interesting arena for exploring the beneficial potential of this alga.

Unbalanced generation of free radicals in the body plays key role in causing many degenerative diseases such as cancer, cardiovascular disease, arthritis and neurodegenerative disorder by damaging cellular DNA, proteins and lipids [7]. Antioxidants are capable of neutralizing free radicals prior to their detrimental physiological effect [8]. Synthetic antioxidants such as butylated hydroxytoluene and butylated hydroxyanisole have recently been reported to have adverse side effects in human health [9, 10]. The search for effective natural antioxidants in food, cosmetic and therapeutic industry is fast emerging as a promising alternative for synthetic antioxidants in respect of low cost, high compatibility with dietary intake and almost zero side effects [5, 11]. Although antioxidant properties of various terrestrial plants are well recognized, corresponding aspects of algae and particularly of *E. tuba* has not been adequately addressed. Therefore the present study is aimed to assess the phytochemical compositions and evaluate antioxidant potential and free radical scavenging activity of a 70% methanol extract of *E. tuba* (ETME).

## Results and discussion

### Algal characterisation

The microscopic observation of cell morphology such as the size of the organism, presence or absence of chloroplast, eyespot, flagella, paramylon bodies etc. were taken into account and identified as *Euglena tuba* (Carter). Microscopic observation of random samples after centrifugation assured that the samples were free from any other algae as well as phytoplankton contaminations which was supported by previous reports that the blooms of Euglena species occur at higher temperature, lower dissolved oxygen and acidic environment with higher nutrient concentration which have significantly inhibit the growth of other algal species and phytoplankton [12–15].

### Phytochemical analysis

**Figure 1:**
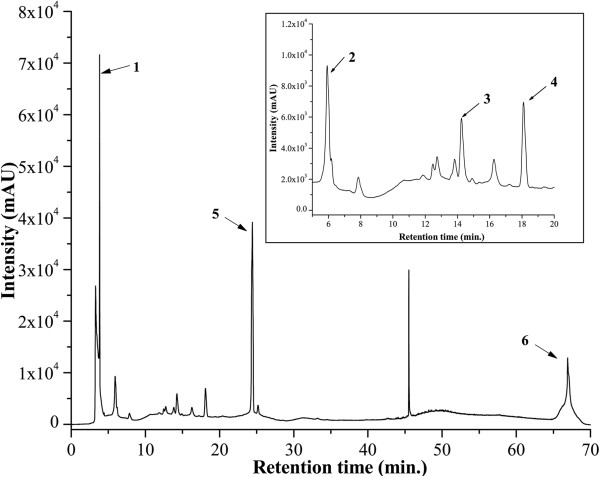
**HPLC chromatogram of ETME.** Inset shows the expanded region of the chromatogram with retention time of 5-20 min. Peaks marked signify the retention peak of (1) tannic acid (3.74), (2) reserpine (5.95), (3) methyl gallate (14.27), (4) catechin (18.08), (5) ascorbic acid (24.48) and (6) rutin (66.96 min) respectively.

Several studies have been made on biological activities of algae and their substance, which could be potential rich sources of natural antioxidants [16]. Das et al. [17] have described the antibacterial potential of the freshwater alga *Euglena viridis* (Ehren). Recently*, Euglena gracilis* Z wild type has already been considered a potential source of vitamin E [18]. Medicinal algae are used as food, flavours, cosmetics, fumigants and insect deterrents, while being the best resources from which novel bioactive compounds are discovered [19]. Qualitative screening of phytochemicals from the algal extract was summarized in Table 1. The results revealed the presence of medically active compounds such as alkaloids, carbohydrates, flavonoids, phenols, saponins, tannins and terpenoids. From Table 1 it was found that the extract contained highest amount of flavonoids, with considerable amounts of alkaloids, phenolics, tannins, reduced carbohydrates and low amount of ascorbic acid content. HPLC analysis was then performed to identify the presence of bioactive compounds by comparing the retention time of reference compounds under the same condition, six main peaks having retention times 3.74, 5.95, 14.27, 18.08, 24.48 and 66.96 min were appeared on the chromatogram of HPLC analysis which were determined to be corresponding to tannic acid, reserpine, methyl gallate, catechin, ascorbic acid and rutin respectively (Figure 1). Most commonly known phytochemicals with antioxidant property are phenolics, flavonoids and tannins which counteract the body’s reactions to allergens, viruses and carcinogens. They show many useful therapeutic roles such as anti-allergic, anti-inflammatory, antimicrobial and anticancer activity [20]. Nitrogen containing organic compounds and alkaloids (such as reserpine) are physiologically active with sedative and analgesic properties and used in relieving pains, anxiety and depression [21]. Glycosides are compounds containing a carbohydrate and non-carbohydrate residue (moiety) in the same molecule. These compounds can be used in the treatment of congestive heart failure and cardiac arrhythmia [20]. Also, the carbohydrates in food are of major interest in relation to chronic diseases. Different types of carbohydrates give rise to different glycemic responses and also able to stimulate lipogenesis [22, 23]. Ascorbic acid, derived from glucose is well established as a naturally occurring antioxidant also reported to attenuate hepatic damage [24]. The obtained results in this study suggested that the identified phytochemical compounds may be the bioactive constituents and this unicellular *Euglena* is proving to be a valuable reservoir of bioactive compounds of substantial medicinal merit.

**Table 1:** **Qualitative and quantitative phytochemical analysis of** ***E. tuba*** **extract**

Tests					Phytochemicals						
	Phen	Flav	Carbo	Tan	Alka	Asc	Ter	Triter	Anth	Sap	Gly
**Qualitative**	+	+	+	+	+	+	+	+	-	+	-
**Quantitative**	11.15 ± 0.001	100.78 ± 2.114	9.99 ± 0.022	5.6 ± 0.047	2.95 ± 0.3	2.16 ± 0.059					

Phen- Phenol, Flav- Flavonoid, Carbo- Carbohydrate, Tan- Tannin, Alka- Alkaloid, Asc-Ascorbic acid, Ter- Terpenoids, Triter- Triterpenoids, Anth- Anthraquinones, Sap- Saponin, Gly- Glycoside; Total phenolics (mg/100 mg extract gallic acid equivalent), Total flavonoids (mg/100 mg extract quercetin equivalent), Carbohydtrate (mg/100 mg extract glucose equivalent), Tannin (mg/100 mg extract catechin equivalent), Alkaloids (mg/100 mg extract reserpin equivalent), Ascorbic acid (mg/100 mg extract ascorbic acid equivalent). “+” Represents presence of the phytoconstituent; “-” represents absence of the phytoconstituent.

### Correlation of antioxidant activity to the phytochemical contents

**Figure 2:**
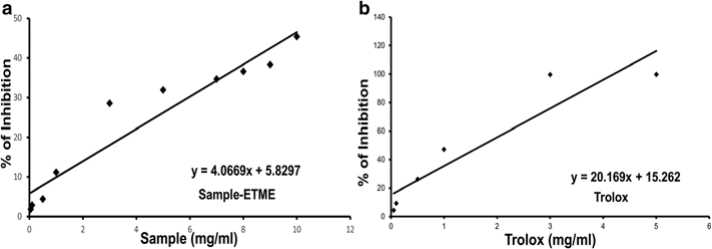
**Total antioxidant activity of ETME (a) and reference compound trolox (b) on ABTS radical cation decolorization assay.** The percentage of inhibition was plotted against concentration of sample. All data are expressed as mean ± S.D. (n = 6).

**Figure 3:**
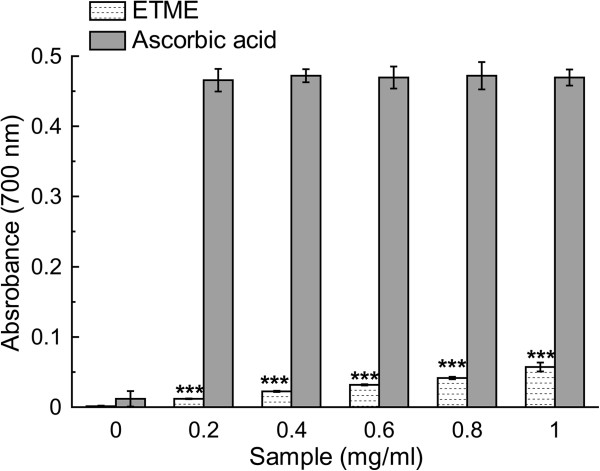
**The reductive ability of the extract and standard ascorbic acid.** The absorbance (700 nm) was plotted against concentration of sample. Each value represents mean ± S.D. (n = 6). ***p <0.001 vs 0 mg/ml.

The antioxidant activity of ETME was measured by using two methods that are complementary to each other, viz., TEAC through ABTS^•+^ radical cation scavenging and reducing power capacity. The assays give an overall picture of the antioxidative ability of the plant extract as summarized in the Figures 2 and 3 along with Table 2. Between the assays, ABTS^•+^ assay is based on interaction between hydrogen‒donating and chain‒breaking antioxidants and which has a characteristic blue colour showing absorption maxima at 734 nm. Interaction with the extract or standard trolox suppressed the absorbance of the ABTS^•+^ radical cation in a dose dependant manner and the results, expressed as percentage inhibition of absorbance, are shown in Figure 2(a) and 2(b) respectively. Previous research has revealed that there is a linear correlation between antioxidant activity and reducing power [25]. The presence of antioxidants in the sample would result in the reducing of Fe^3+^ to Fe^2+^ by donating an electron. As illustrated in Figure 3, Fe^3+^ to Fe^2+^ transformation in the presence of the extract and reference ascorbic acid was found increasing with increasing concentrations. Although the activity of ascorbic acid was better than the sample, the latter showed significantly low reducing capability which may serve as a significant indicator of its antioxidant potential.

**Table 2:** **Comparison of the antioxidant and free radical scavenging capacities of 70% methanol extract of** ***E. tuba*** **and standard reference compounds**

Name of assay	ETME	Standard	Values of standard compounds
TEAC Values	0.202 ± 0.001	**-**	**-**
*IC_50_ values of the extracts for free radical scavenging capacity for			
DPPH	146.07 ± 1.80	Ascorbic acid	5.29 ± 0.28^***^
Hydroxyl radical (OH^•^) scavenging	41.89 ± 0.41	Mannitol	571.45 ± 20.12^***^
Lipid peroxidation	202.49 ± 33.32	Trolox	6.76 ± 0.17^***^
Superoxide anion (O_2_^•-^) scavenging	5.83 ± 0.07	Quercetin	42.06 ± 1.35^***^
Hydrogen peroxide (H_2_O_2_) scavenging	47.34 ± 5.05	Sodium pyruvate	3.24 ± 0.30^***^
Nitric oxide radical (NO) scavenging	278.46 ± 15.02	Curcumin	90.82 ± 4.75^***^
Peroxynitrite (ONOO^-^) scavenging	2.821 ± 1.69	Gallic acid	0.876 ± 0.57^***^
Singlet oxygen (^1^O_2_) scavenging	0.879 ± 0.29	Lipoic acid	0.046 ± 0.01^***^
Hypochlorous acid (HOCl) scavenging	223.25 ± 4.19	Ascorbic acid	235.96 ± 5.75^**^

*IC_50_ Values are in μg/ml (except for Hydrogen peroxide, Peroxynitrite and Singlet oxygen scavenging assay where values are expressed in mg/ml). Each value represents mean ± S.D. (n = 6). ***p < 0.001, **p < 0.01 vs ETME.

**Figure 4:**
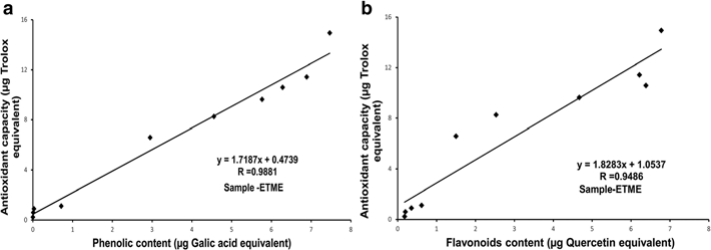
**Correlation of total antioxidant activity with phenolic and flavonoid contents.** The relationship between **(a)** total phenolic content or **(b)** total flavonoid content in the extract and their antioxidant capacity. The correlation analyses were described as linear correlation coefficient (R).

Since the antioxidant property of natural antioxidants is generally attributed to their phytochemical constituents, TEAC value of ETME was correlated to its total phenolic and flavonoid content. As shown in Figure 4(a), the total phenolic content of ETME significantly correlated with antioxidant activity (correlation coefficient *R* = 0.9881), which proved that the phenolic content of ETME highly ascertained the antioxidant activity. Alongside, the correlation coefficient of ETME for flavonoid content with its antioxidant capacity was highly significant (correlation coefficient *R* = 0.9486), correlated with their antioxidant activity (Figure 4(b)).

### DPPH radical scavenging assay

**Figure 5:**
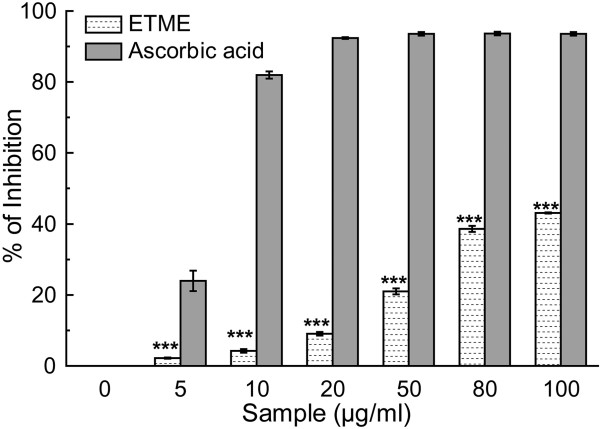
**DPPH radical scavenging activity of ETME and standard ascorbic acid.** Each value represents mean ± S.D. (n = 6). ***p < 0.001 vs 0 μg/ml.

DPPH free radical is commonly used to determine radical scavenging activity of natural compounds due to its stable nature. DPPH in its radical form absorbs at 517 nm, but upon reduction with an antioxidant, its absorption decreases due to the formation of its non‒radical form, DPPH–H [26]. Thus, the radical scavenging activity in the presence of a hydrogen donating antioxidant can be monitored as a decrease in absorbance of DPPH solution. ETME showed excellent dose-dependent scavenging activity of DPPH radical (Figure 5) but lower than the standard ascorbic acid. The IC_50_ values (Table 2) of the extract and standard ascorbic acid were 146.07 ± 1.80 μg/ml and 5.29 ± 0.28 μg/ml respectively.

### Scavenging of Reactive Oxygen Species (ROS)

**Figure 6:**
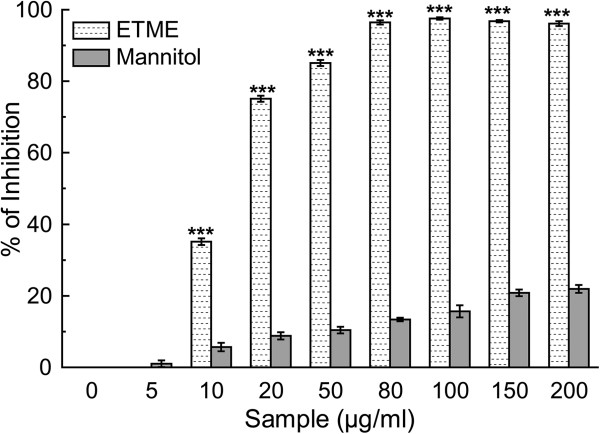
**Hydroxyl radical scavenging activity of the ETME and the reference compound mannitol.** The data represent the percentage of inhibition of deoxyribose degradation. The results are mean ± S.D. (n = 6). ***p < 0.001 vs 0 μg/ml.

**Figure 7:**
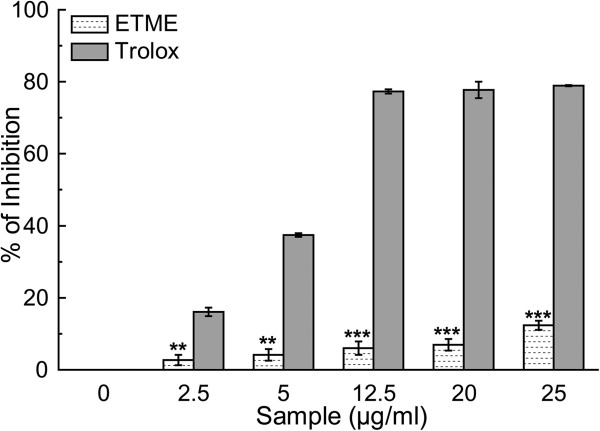
**The inhibitory effect of ETME and standard trolox on lipid peroxidation.** This phenomenon is shown as percentage inhibition of peroxidation of lipids from mice brain homogenate. The results are mean ± S.D. (n = 6). **p < 0.01 and ***p < 0.001 vs 0 μg/ml.

Hydroxyl radical, the most detrimental ROS induces several damages to DNA, lipids and proteins [27]. It can also accelerate lipid peroxidation by decomposing lipid hydroperoxides into proxy and alkoxyl radicals that can perpetuate the chain reaction [28]. The ability of the extract to inhibit hydroxyl radical-mediated deoxyribose degradation in a Fe^3+^-EDTA-ascorbic acid and H_2_O_2_ reaction mixture was exhibited in hydroxyl radical scavenging assay. The results, as can be found from Figure 6 and Table 2, indicate that the extract is more effective hydroxyl radical scavenger than standard mannitol. Similar physiological damage of the liposomes by OH^•^ radical induces lipid peroxidation via pathways involving iron and singlet oxygen by the Fenton reaction (H_2_O_2_ + Fe^2+^ = Fe^3+^ + OH^-^ + OH^•^) [29]. Lipo-peroxidation inhibitory effect of the extract is reflected in Figure 7 in a dose dependent manner, similarly like standard trolox, although the calculated IC_50_ values for the extract in comparison to that of trolox (Table 2) indicate that the sample is not as potent inhibitor of lipid peroxidation as the reference compound.

**Figure 8:**
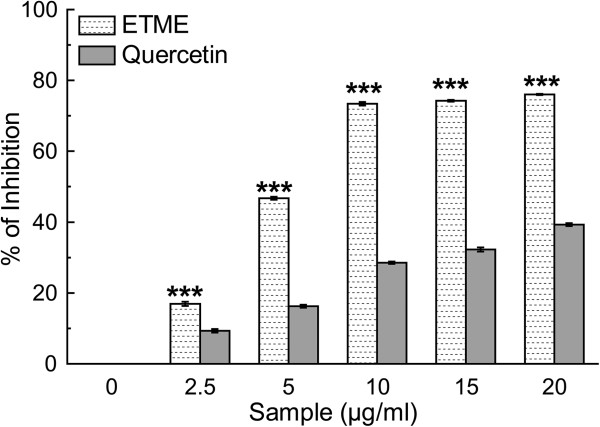
**Scavenging effect of** ***E. tuba*** **extract and standard quercetin on superoxide radical.** The data represents the percentage of superoxide radical inhibition. All data are expressed as mean ± S.D. (n = 6). ***p < 0.001 vs 0 μg/ml.

Superoxide anion radical is well known as an initiator radical in the formation of other reactive oxygen-species, such as hydrogen peroxide or singlet oxygen in living systems [30]. The decrease in absorbance at 560 nm with the extract and the reference compound quercetin indicate their abilities to quench superoxide radicals in the reaction mixture (Figure 8). The IC_50_ values of the same (Table 2) indicate that the extract is more potent superoxide radical scavenger than standard compound quercetin.

**Figure 9:**
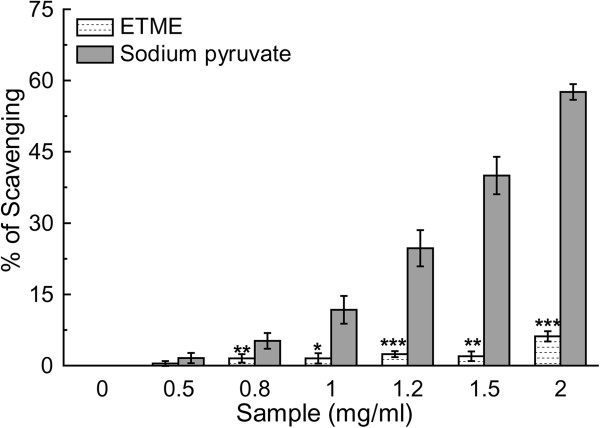
**H**_**2**_ **O**_**2**_ **scavenging assay of ETME and sodium pyruvate.** Values are expressed as mean ± S.D. (n = 6). *p < 0.05, **p < 0.01 and ***p < 0.001 vs 0 mg/ml.

Hydrogen peroxide, a weak oxidizing agent, can cross biological membranes and involve in the generation of hydroxyl radicals. This property has placed hydrogen peroxide in a more prominent role to initiate cytotoxicity than its chemical reactivity. Thus removing H_2_O_2_ is very important for the protection of living systems [31]. The plot of the scavenging activities in a concentration-dependent manner of the extract compared to standard sodium pyruvate (Figure 9) shows that the former is a mediocre scavenger of H_2_O_2_, as is also reflected from their respective IC_50_ values (Table 2). Scavenging of H_2_O_2_ by extracts may be attributed to their polyphenolics, which can donate electrons to H_2_O_2_, thus neutralizing it to water [32].

**Figure 10:**
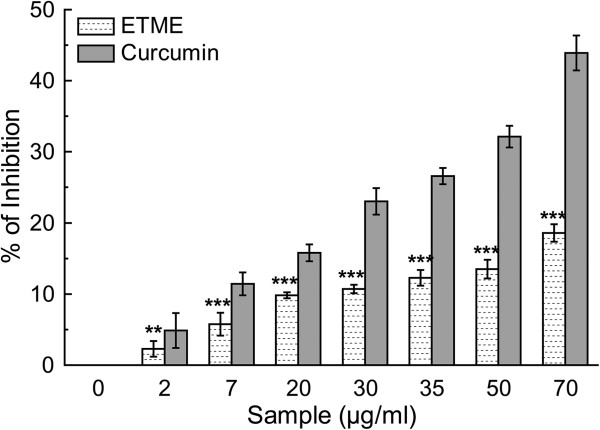
**The nitric oxide radical scavenging activity of** ***E. tuba*** **extract and standard curcumin.** The data represents the % of nitric oxide inhibition. Each value represents mean ± S.D. (n = 6). ***p < 0.001 vs 0 μg/ml.

**Figure 11:**
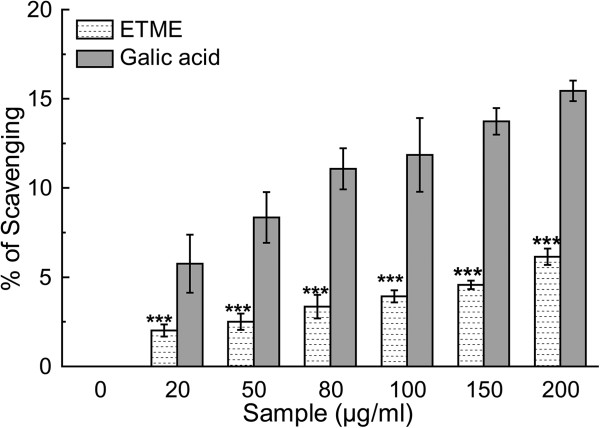
**Peroxynitrite anion scavenging assay of** ***E. tuba*** **extract and the standard gallic acid.** Value represents mean ± S.D. (n = 6). ***p < 0.001 vs 0 μg/ml.

Nitric oxide is well known to have an important role in various inflammatory processes. The toxicity of NO increases greatly when it reacts with superoxide radical, forming the highly reactive peroxynitrite anion (ONOO^-^) [33]. The extract inhibits the nitrite formation by directly competing with oxygen in the reaction with nitric oxide. The results (Figure 10 and Table 2) show that ETME has been found to be efficient in scavenging nitric oxide in a dose dependent manner, although not as potent as standard curcumin. Protonated peroxynitrite (ONOO^-^) forms highly reactive peroxynitrous acid (ONOOH) and generate excess ONOO^-^ which leads to oxidative damage and tissue injury [27]. When different amounts of extract and standard reference compound gallic acid were added to the reaction mixture, ONOO^-^**-**mediated oxidising activity was inhibited markedly in a concentration-dependent manner, as shown in Figure 11 even not as well as the reference compound (Table 2).

**Figure 12:**
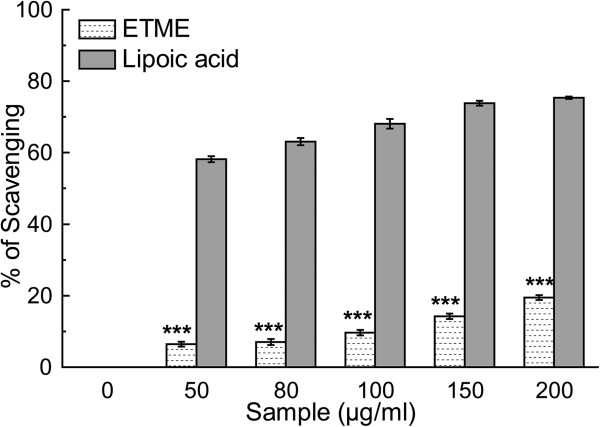
**Effect of ETME and standard lipoic acid on the scavenging of singlet oxygen.** The results are mean ± S.D. (n = 6). ***p < 0.001 vs 0 μg/ml.

Singlet oxygen is a high energy form of oxygen generally produced in biological systems by photoexcitation upon exposure to UV radiation or by chemiexcitation. It induces hyper-oxidation, oxygen cytotoxicity and decreases the antioxidant activity [34]. Figure 12 and the IC_50_ values from Table 2 illustrate the ability of scavenging singlet oxygen by the extract and standard lipoic acid.

**Figure 13:**
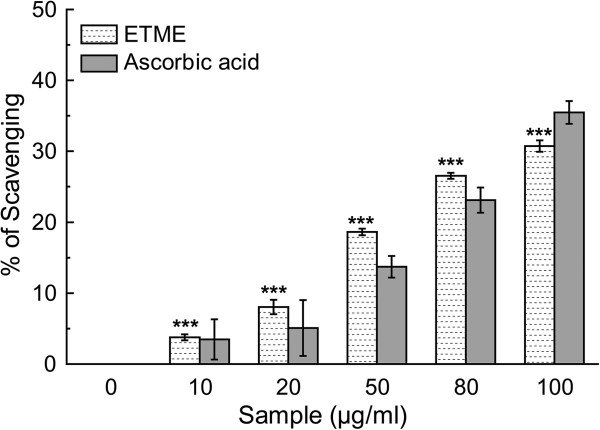
**Hypochlorous acid scavenging activity of** ***E. tuba*** **extract and standard ascorbic acid.** All data are expressed as mean ± S.D. (n = 6). ***p < 0.001 vs 0 μg/ml.

HOCl is produced *in vivo* by the oxidation of Cl^-^ ions catalysed by neutrophil-derived myeloperoxidase in the presence of H_2_O_2_, at sites of inflammation [35]. HOCl damages and induce target cell lysis, caused by sulfhydryl oxidation in plasma membrane proteins and inactivates antioxidant enzymes like catalase [36]. Thus, this extract may have protective effects *in vivo* during inflammation processes. Dose-dependent hypochlorous acid scavenging activity of ETME and standard ascorbic acid was found in this study as shown in Figure 13 and the IC_50_ values (Table 2).

## Conclusions

Literature survey revealed that *Ginkgo biloba, Curcuma longa, Spondias pinnata* and several other plants were confirmed as a source for potent antioxidant phytochemicals due to scavenging activity of the similar free radicals [27, 37, 38] as ETME. Considering the results obtained, it may be anticipated that 70% methanol extract of *E. tuba*, which contains large amounts of bioactive phytocompounds, exhibits high antioxidant and free radical scavenging activities with high reducing power capacity. Scavenging abilities of the extract was observed mainly on superoxide, hydroxyl and hypochlorous acid radicals. These *in vitro* assays indicate that this algal extract is a significant source of natural antioxidant, which might be helpful in preventing the progress of various oxidative stresses which is also beneficial in prevention of “various other human diseases” [39]. However, the *in vivo* antioxidant activity of this extract needs to be assessed prior to clinical use.

## Methods

### Chemicals

2,2′-azinobis-(3-ethylbenzothiazoline-6-sulfonic acid) (ABTS) was procured from Roche diagnostics, Mannheim, Germany, and 6-hydroxy-2,5,7,8-tetramethylchroman-2-carboxylic acid (Trolox) was obtained from Fluka, Buchs, Switzerland. Potassium persulfate (K_2_S_2_O_8_), 2-deoxy-2-ribose, ethylene diammine tetraacetic acid (EDTA), ascorbic acid, trichloroacetic acid (TCA), mannitol, nitro blue tetrazolium (NBT), reduced nicotinamide adenine dinucleotide (NADH), phenazine methosulfate (PMS), sodium nitroprusside (SNP), 1,10-phenanthroline, sulphanilamide, N-(1-Naphthyl)ethylenediamine dihydrochloride (NED), L-histidine, lipoic acid, sodium pyruvate, quercetin and ferrozine were obtained from Sisco Research Laboratories Pvt. Ltd, Mumbai, India. Hydrogen peroxide, potassium hexacyanoferrate, Folin-ciocalteu reagent, sodium carbonate, mercuric chloride, potassium iodide, anthrone, vanillin, thiourea, 2,4-dinitrophenylhydrazine, sodium hypochlorite, aluminium chloride, xylenol orange, butylated hydroxyltoluene (BHT) and N,N- dimethyl-4-nitrosoaniline were procured from Merck, Mumbai, India. 1,1-diphenyl-2-picrylhydrazyl (DPPH), gallic acid were obtained from MP Biomedicals, France. Catalase and sodium bicarbonate were obtained from HiMedia Laboratories Pvt. Ltd, Mumbai, India. Evans Blue was purchased from BDH, England. D-glucose was procured from Qualigens Fine Chemicals, Mumbai. Diethylenetriaminepentaacetic acid (DTPA) was obtained from Spectrochem Pvt. Ltd, Mumbai, India. Thiobarbituric acid (TBA) was obtained from Loba Chemie, Mumbai, India.

### Algal characterisation and extract preparation

Algal samples were collected with the help of a plankton net made of bolting silk cloth from different ponds of ‘Cachar’ district of the state of Assam, India, situated at N24°41′23.64″ E92°45′04.42″ and 36.5 MSL. The samples were preserved in formalin (4%) for identification and observed under the light microscope following standard keys [40, 41]. Simultaneously, fresh algal samples were thoroughly cleaned in sterile distilled water for three times to remove extraneous materials adhering to it and finally centrifuged at 1000 × *g* using macro rotor to remove contaminated bacteria [17]. The collected pellet as biomass of euglena was dried at ambient temperature for seven days, finely powdered for extraction purpose. The powder (100 g) was mixed with 1000 ml methanol:water (7:3) using a magnetic stirrer for 15 h and then centrifuged at 2850× *g* for obtaining the supernatant. The process was repeated by mixing the precipitated pellet with 1000 ml fresh solvent. The supernatants from both the phases were mixed and concentrated under reduced pressure in a rotary evaporator, followed by lyophilisation. The lyophilized 70% methanol extract of *E. tuba*, designated as ETME was kept at -20°C for future use. A freshly prepared aqueous solution of ETME of different concentration was used for various experiments.

### Animals

Adult male Swiss Albino mice (*Mus musculus)* weighing 20-25 g, required for lipid peroxidation inhibition study were kept as 4 mice/cage at 25 ± 2°C and 60 ± 5% humidity and normal photo cycle (12 h dark/12 h light), and supplied with *ad libitum* laboratory diet and water. The Institutional Animal Ethics Committee of the Institute (Registration number: 95/1999/CPCSEA) approved use of the animals for experimentation.

### Phytochemical analysis

#### Qualitative analysis

Phytochemical analysis of ETME was carried out using standard qualitative methods by Harborne and Baxter [42] and Kokate et al. [43]. The components analysed for phytochemicals were alkaloids, carbohydrates, flavonoids, glycosides, phenols, saponins, tannins, terpenoids, anthraquinones and triterpenoids.

### Quantitative phytochemical analysis

#### Total phenolic content

The total phenolic content present in ETME was determined using Folin-Ciocalteu (FC) method done earlier [27]. Briefly, 0.1 ml extract was mixed with 0.75 ml FC reagent (previously diluted 1000-fold with distilled water), followed by the addition of 0.06% Na_2_CO_3_ (0.75 ml) solution. After incubation at 22°C for 90 min, the absorbance was taken at 725 nm. All tests were performed six times. The phenolic content was evaluated from a gallic acid standard curve.

#### Total flavonoid content

Total flavonoid content was determined according to Hazra et al. [27]. 0.1 ml extract was added to 0.03 ml 5% NaNO_2_. After incubation for 5 min at 25°C, AlCl_3_ (0.03 ml, 10%) was added, followed by 0.2 ml 1 mM NaOH. Finally, the reaction mixture was diluted to 1 ml with water and the absorbance was measured at 510 nm. The flavonoid content from six repetitions was calculated from a quercetin standard curve.

### Quantification of carbohydrate content

Carbohydrate content of the extract was quantified following previously performed method [44]. Briefly, 100 mg of ETME was hydrolysed with 5 ml of 2.5 N HCl. The mixture was diluted to 100 ml with distilled water and centrifuged. 0.25 ml supernatant was made up to 0.5 ml using distilled water and mixed with 4 ml anthrone reagent and was incubated at 95°C for 8 min. After incubation, absorbance of the resultant dark green coloured solution was measured at 630 nm. All tests were performed six times. The carbohydrate content was evaluated from a glucose standard curve.

### Quantification of alkaloid content

Quantification of alkaloid content of ETME was done using previously implemented protocol [45]. To 1 ml of extract (1 mg/ml) in water 0.1 ml of FeCl_3_ (2.5 mM FeCl_3_ in 0.5 M HCl) was added followed by addition of 0.1 ml 1,10-phenanthroline. After incubation for 30 min at 70°C the absorbance was measured at 500 nm. All tests were performed six times. The alkaloid content was quantified from the reserpine standard graph.

### Quantification of ascorbic acid content

Ascorbic acid content quantification was accomplished according to the previously elucidated technique [45]. In brief, 1 ml aliquots of ETME (1 mg/ml) in water were mixed with 1 ml of ‘2,4-dinitro-phenylhydrazine reagent’ and was incubated at 95°C for 15 min. After incubation, 5 ml of 85% H_2_S0_4_ was added drop wise to the reaction mixture in ice cold condition. After 30 min, the absorbance was measured at 520 nm. All tests were performed six times. The ascorbic acid content was expressed as L-ascorbic acid equivalent.

### Quantification of Tannin content

This assay was performed according to the technique followed earlier [44]. Briefly, 0.1 ml aliquot of ETME (1 mg/ml) was mixed with the 0.5 ml vanillin hydrochloride reagent and incubated for 20 min at room temperature. The absorbance of the resulting magenta-pink colour was measured at 500 nm. All tests were performed six times. The tannin content was evaluated from a catechin standard graph.

### HPLC standardisation of the extract

For HPLC analysis, stock solutions (10 μg/ml) were prepared in the mobile phase for the sample and different standard phytocompounds. Samples were then filtered through 0.45 μm polytetrafluoroethylene (PTFE) filter (Millipore) to remove any particulate matter. Analysis was performed using a HPLC-Prominence System RF10AXL (Shimadzu Corp., Japan) equipped with degasser (DGU-20A_5_), quaternary pump (LC-20AT), auto-sampler (SIL-20A) and detectors of Reflective Index (RID-10A), Fluorescence (RF-10AXL) and Diode Array (SPD-M20A). The injection volume used was 20 μl and the sample and standards were analyzed in triplicates. Gradient elution consecutive mobile phases of acetonitrile and 0.5 mM ammonium acetate in water, at a flow rate of 1 ml/min for 80 min through the column (ZIC®-HILIC) that was maintained at 25°C. The detection was carried out at 254 nm.

### In vitro antioxidant and free radical scavenging assays

#### Total antioxidant activity

Antioxidant capacity of the extract (0.05-10 mg/ml) were evaluated by ABTS^•+^ radical cation decolourisation assay in comparison to trolox standard [27]. The absorbance of the ABTS^•+^ solution was equilibrated to 0.70 (± 0.02) by diluting with water at room temperature, then 1 ml ABTS^•+^ solution was mixed with 10 μl of the test sample and the absorbance was measured at 734 nm after 6 min. The experiment was repeated six times. The percentage inhibition of absorbance was calculated and plotted as a function of the concentration of standard and sample to determine the trolox equivalent antioxidant concentration (TEAC), calculated as the ratio of the gradients of the plots for the sample to trolox.

#### Measurement of reducing power

The Fe^3+^-reducing power of the extract was determined by a standard method [27]. Different concentrations (0–1.0 mg/ml) of the extract were mixed with equivolume 0.2 M phosphate buffer (pH 6.6) and 0.1% potassium hexacyanoferrate, followed by incubation for 20 min at 50°C. After incubation, the reaction was terminated with 0.5 ml 10% TCA. Then, 1 ml reaction mixture was diluted with 1 ml distilled water followed by the addition of 0.1 ml FeCl_3_ solution (0.01%). The reaction mixture was left for 10 min at room temperature and the absorbance was measured at 700 nm against an appropriate blank solution. All tests were performed six times. Ascorbic acid was used as a standard.

#### DPPH radical scavenging assay

The complementary study for the antioxidant capacity of the extract was confirmed by the DPPH (1,1-diphenyl-2-picrylhydrazyl) scavenging assay according to Mahakunakorn et al. [46], with slight modification. Different concentrations (0-100 μg/ml) of the extract and the standard ascorbic acid were mixed with equal volume of ethanol. Then 50 μl of DPPH solution (1 mM) was added into the mixture and stirred thoroughly. The resulting solution was kept standing for 2 min before the OD was measured at 517 nm. The measurement was repeated with six sets. The percentage of scavenging was calculated from the values of the control and the test samples.

#### Hydroxyl radical scavenging assay

The hydroxyl radical scavenging assay was performed using a standard protocol [27], based on quantification of the degradation product of 2-deoxyribose condensed with TBA. Hydroxyl radical was generated by the Fe^3+^-ascorbate-EDTA-H_2_O_2_ system (the Fenton reaction). In a final volume of 1 ml, various concentrations of the test sample or reference compound was mixed with 2-deoxy-2-ribose (2.8 mM); KH_2_PO_4_-KOH buffer (20 mM, pH 7.4); FeCl_3_ (100 μM); EDTA (100 μM); H_2_O_2_ (1.0 mM); ascorbic acid (100 μM) and incubated for 1 h at 37°C. 0.5 ml of the reaction mixture was added to 2.8% TCA, followed by 1% TBA and incubated at 90°C to develop the colour of TBARS (Thiobarbituric acid reactive substance). Absorbance was measured at 532 nm against an appropriate blank solution. All tests were performed six times. Mannitol, a classical OH^•^ scavenger, was used as a positive control. Percentage inhibition was evaluated by comparing the test and blank solutions.

#### Inhibition of lipid peroxidation

The ability of ETME to inhibit lipid peroxidation was evaluated by the method of Sarkar et al. [47]. Brain homogenate was prepared by centrifuging Swiss Albino mice brain with 50 mM phosphate buffer and 120 mM KCl. An aliquot of the supernatant homogenate was mixed with various concentrations of the extract (2.5-25 μg/ml) along with the standard Trolox, followed by addition of 0.1 mM FeSO_4_ and 0.1 mM ascorbic acid and incubated for 1 h at 37°C to generate TBARS. After stopping the reaction with TCA, TBA was added and the absorbance of the supernatant was taken at 532 nm. All tests were repeated six times.

#### Superoxide radical scavenging assay

This activity was measured by the reduction of NBT according to an earlier method [48]. The 1 ml reaction mixture contained phosphate buffer (20 mM, pH 7.4), NADH (73 μM), NBT (50 μM), PMS (15 μM) and various concentrations (0-20 μg/ml) of sample and standard quercetin solution. After incubation for 5 min at ambient temperature (20-25°C), quantity of generated formazan was measured at 562 nm against an appropriate blank. All tests were performed six times.

#### Hydrogen peroxide scavenging assay

FOX-reagent was used to evaluate the H_2_O_2_ scavenging property of ETME with reference to sodium pyruvate [27]. An aliquot of 50 mM H_2_O_2_ and various concentrations (0-2 mg/ml) of samples were mixed (1:1 v/v) and incubated for 30 min at room temperature. 90 μl of the incubated reaction mixture was mixed with 10 μl HPLC-grade methanol followed by 0.9 ml FOX reagent and incubated at ambient temperature for 30 min. The absorbance of the ferric-xylenol orange complex was measured at 560 nm. All tests were carried out six times.

#### Nitric oxide radical scavenging assay

Nitric oxide generated from the SNP aqueous solution at physiological pH, interacts with oxygen to produce nitrite ions which were measured by Griess Illosvoy reaction [27]. The reaction mixture (3 ml) contained 10 mM SNP, phosphate buffered saline (pH 7.4) and various doses of ETME (0-70 μg/ml). Curcumin was used as a standard compound. After incubation for 150 min at 25°C, 1 ml sulfanilamide (0.33% in 20% glacial acetic acid) was added to 0.5 ml of the incubated solution and again after 5 min, 1 ml NED (0.1% w/v) was mixed and incubated for 30 min at 25°C. The pink chromophore generated was measured spectrophotometrically at 540 nm against a blank sample. All tests were performed six times.

#### Peroxynitrite radical scavenging assay

Peroxynitrite (ONOO^-^) was synthesized 12 h before the experiment as described by Beckman et al. [49]. The peroxynitrite scavenging activity of the extract and reference gallic acid was measured by Evans’ blue bleaching assay according to a standard method [27]. In a 1 ml reaction mixture contained 50 mM phosphate buffer (pH 7.4), 0.1 mM DTPA, 90 mM NaCl, 5 mM KCl, 12.5 μM Evans Blue, various doses ETME (0–200 μg/ml) and 1 mM peroxynitrite. After incubation at 25°C for 30 min the absorbance was measured at 611 nm. The percentage scavenging of ONOO^-^ was calculated by comparing the results of the test and blank samples. All tests were performed six times.

#### Singlet oxygen radical scavenging assay

The production of singlet oxygen (^1^O_2_) was determined by monitoring N,N-dimethyl-4-nitrosoaniline (RNO) bleaching, using a previously reported protocol [27]. The final reaction mixture (2 ml) contained 45 mM phosphate buffer (pH 7.1), 50 mM NaOCl, 50 mM H_2_O_2_, 50 mM histidine, 10 μM RNO and various concentrations (0-200 μg/ml) of sample. After incubation at 30°C for 40 min the decrease in RNO absorbance was measured at 440 nm. The scavenging activity of sample was compared with that of a reference compound, lipoic acid. All tests were performed six times.

#### Hypochlorous acid scavenging assay

According to a standard protocol described by Hazra et al. [27] the 1 ml reaction mixture contained 50 mM phosphate buffer (pH 6.8), catalase (7.2 μM), freshly prepared HOCl (8.4 mM) and increasing concentrations (0-100 μg/ml) of ETME. The assay mixture was incubated at 25°C for 20 min and the scavenging activity of the extract and the standard ascorbic acid was evaluated by measuring the decrease in absorbance of catalase at 404 nm.

#### Statistical analysis

All data are given as the mean ± SD of six measurements. Statistical analysis was performed using KyPlot version 2.0 beta 15 (32 bit). The IC_50_ values were calculated by the formula Y = 100*A1/(X + A1), where A1 = IC_50_, Y = response (Y = 100% when X = 0), X = inhibitory concentration. The IC_50_ values were compared by paired ‘*t*’ tests. P < 0.05 was considered significant. All the graphics were finally processed using Adobe Photoshop v.7.
